# Associations between excessive fatigue and pain, sleep, mental-health and work factors in Norwegian nurses

**DOI:** 10.1371/journal.pone.0282734

**Published:** 2023-04-04

**Authors:** Stand Hiestand, Ingeborg Forthun, Siri Waage, Ståle Pallesen, Bjørn Bjorvatn

**Affiliations:** 1 Department of Global Public Health and Primary Care, University of Bergen, Bergen, Norway; 2 Department of Disease Burden, Norwegian Institute of Public Health, Bergen, Norway; 3 Norwegian Competence Center for Sleep Disorders, Haukeland University Hospital, Bergen, Norway; 4 Department of Psychosocial Science, University of Bergen, Bergen Norway; University of Rijeka Faculty of Medicine: Sveuciliste u Rijeci Medicinski fakultet, CROATIA

## Abstract

**Aim:**

To investigate whether pain, sleep duration, insomnia, sleepiness, work-related factors, anxiety, and depression associate with excessive fatigue in nurses.

**Background:**

Fatigue among nurses is a problem in the context of ongoing nursing shortages. While myriad factors are associated with fatigue not all relationships are understood. Prior studies have not examined excessive fatigue in the context of pain, sleep, mental health, and work factors in a working population to determine if associations between excessive fatigue and each of these factors remain when adjusting for each other.

**Methods:**

A cross-sectional questionnaire study among 1,335 Norwegian nurses. The questionnaire included measures for fatigue (Chalder Fatigue Questionnaire, score ≥4 categorized as excessive fatigue), pain, sleep duration, insomnia (Bergen Insomnia Scale), daytime sleepiness (Epworth Sleepiness Scale), anxiety and depression (Hospital Anxiety and Depression Scale), and work-related factors. Associations between the exposure variables and excessive fatigue were analyzed using chi-square tests and logistic regression analyses.

**Results:**

In the fully adjusted model, significant associations were found between excessive fatigue and pain severity scores for arms/wrists/hands (adjusted OR (aOR) = 1.09, CI = 1.02–1.17), hips/legs/knees/feet (aOR = 1.11, CI = 1.05–1.18), and headaches/migraines (aOR = 1.16, CI = 1.07–1.27), sleep duration of <6 hours (aOR = 2.02, CI = 1.08–3.77), and total symptom scores for insomnia (aOR = 1.05, CI = 1.03–1.08), sleepiness (aOR = 1.11, CI = 1.06–1.17), anxiety (aOR = 1.09, CI = 1.03–1.16), and depression (aOR = 1.24, CI = 1.16–1.33). The musculoskeletal complaint-severity index score (aOR = 1.27, CI = 1.13–1.42) was associated with excessive fatigue in a separate model adjusted for all variables and demographics. Excessive fatigue was also associated with shift work disorder (OR = 2.25, CI = 1.76–2.89) in a model adjusted for demographics. We found no associations with shift work, number of night shifts and number of quick returns (<11 hours between shifts) in the fully adjusted model.

**Conclusion:**

Excessive fatigue was associated with pain, sleep- and mental health-factors in a fully adjusted model.

## Introduction

One way to define fatigue is, “The awareness of a decreased capacity for physical and/or mental activity due to an imbalance in the availability, utilization, and or restoration of resources needed to perform activity,” [1]. However, fatigue’s definition is not clearly agreed upon due to its complex and multidimensional nature [2]. Models of fatigue are many and vary from the neuromuscular [3] to biomathematical predictive models [4]. Fatigue may have physical/muscular [5] and/or mental components [6]. It may be acute or chronic [7], or warrant the diagnosis chronic fatigue syndrome [8]. In real world situations, it may be difficult to parse physical and mental fatigue, therefore for the purposes of this paper we examine fatigue using both physical and mental components together.

Fatigue has been listed as the number one condition associated with productivity loss at work and the third most costly when considering medical, drug and productivity costs together [9]. Fatigue’s estimated productivity loss costs $136.4 billion annually in the U.S. [10]. It is a common condition, reported by as many as one in five individuals [11] and one in four patients [12]. In a general adult population sample in Norway, fatigue was the most frequently reported symptom, 59.7% of participants reported some type of fatigue [13]. Worldwide, there is a 10% prevalence of chronic fatigue and a 1% prevalence of chronic fatigue syndrome [14].

Nursing shortages have long been a problem and have been referred to as a global crisis since 2002 [15]. As of 2018 there was an estimated shortfall of 5.9 million nurses worldwide, and a high proportion of nurses nearing retirement further threatens nursing workforce numbers [16]. It is thus vital to identify actionable and modifiable factors related to fatigue in nurses, as these can inform preventive and other measures to retain nurses.

Myriad factors associate with fatigue, but not all the relationships are fully understood. It is fairly well-documented that women have an increased risk of fatigue compared to men [17–21]. However, there are examples of studies that found no significant association between sex and excessive/pathological fatigue [22, 23]. Research on the relationship between fatigue and age is also inconsistent. It has been reported that older age is associated with greater fatigue [24, 25], that there is no statistically significant association [20, 22, 23] or that there is lower general and/or mental fatigue in older individuals [17–19]. A Danish study indicates that the risk of general fatigue significantly decreases as age increases for healthy adults, but that an inverse relationship exists for those with somatic disease [26].

Pain associates with fatigue in conditions like lupus [27], cancer [28] and arthritis [29], but research on its association with fatigue in general/working populations is limited and demands further exploration. A Dutch community study [30] and a study on Iranian office workers [31] both indicated associations between fatigue and pain. A Brazilian study, however, found no significant association between fatigue and musculoskeletal discomfort [32].

Persistent fatigue has been reported in 37% of community individuals with chronic regional pain compared to 47% - 64% of those with chronic widespread pain [30]. Therefore, clear associations between fatigue and pain in multiple regions together are potentially more likely than pain in individual regions. However, it is possible that pain in some regions may contribute to fatigue more than pain in others and identifying those regions may be of use for future interventions. Thus, measuring both pain by region and as a composite may be of use.

Sleep deprivation [33], poor sleep quality [17, 18, 34], insomnia and a wide range of other sleep disorders [35] are associated with fatigue. There may also be a relationship between excessive sleepiness and fatigue, although that is less clear. In a Canadian study on sleep-disordered patients, the majority of those with sleepiness also reported fatigue, whereas the majority of those with pathological fatigue did not report sleepiness [36]. Whereas, in a U.S study, daytime sleepiness in nurses was significantly associated with occupational fatigue [17]. Further, shift work disorder (SWD, a sleep disorder related to one’s work schedule that is characterized by sleepiness and insomnia) correlated positively with fatigue in crude but not adjusted analyses [37].

Depression and anxiety [38, 39] clearly associate with fatigue. Workplace factors that correlate with fatigue or chronic fatigue include quick returns (<11h between shifts) [22, 23, 40, 41], night work [40, 42] and shift work [21, 43].

The relationships between fatigue and pain, sleep factors, mental health and work-related factors may be complicated by the relationships these factors have with each other. For example, pain has also been associated with insomnia [44, 45], excessive sleepiness [44] and quick returns [45]. And, in a study on sick leave for chronic back pain, those reporting substantial fatigue had both higher intensity of pain and more symptoms of depression than those without substantial fatigue [46]. Pain, anxiety and depression, sleep factors and work factors are thus entwined in a complex web but are still often studied as discrete/incomplete entities. Even in a recent comprehensive paper on occupational fatigue that includes personal and work factors [17], depression, anxiety, insomnia and quick returns are not investigated despite findings showing that all these factors may explain or exacerbate fatigue in nurses. It is therefore of importance to determine if pain, sleep/sleep-disorders, mental-health and work variables associate with fatigue when they are controlled for the presence of each other as well as demographic factors.

The present study aims to determine the associations between excessive fatigue and pain, sleep factors, mental health, and work-related factors after adjusting for all variables in the model. To our knowledge this is the first study examining all these variables together in a working population. Our hypotheses were as follows: Compared to their counterparts; Nurses with greater pain severity will more likely have excessive fatigue (H1), nurses with sleep problems will more likely have excessive fatigue (H2), nurses with high levels of anxiety and depressive symptoms will more likely have excessive fatigue (H3), nurses with shift work, night work and quick returns will more likely have excessive fatigue (H4).

## Methods

### Study design, settings and participants

Data stemmed from The SUrvey of Shift work, Sleep and Health (SUSSH), an ongoing cohort study of Norwegian nurses. Initial data collection was conducted from December 2008 to March 2009 when 6,000 randomly selected nurses were invited to participate. The random sample was drawn from the Norwegian Nurses Organization’s membership list using five strata categorized by time elapsed since graduation (less than 12 months, 1–3 years, >3–6 years, >6–9 years and >9–12 years). Everyone in the sample received an invitation to join the study and a questionnaire via postal mail. They were asked to return completed questionnaires in a pre-paid envelope. Responders were entered in a lottery with a prize of a 500 NOK gift card. Six hundred of the initial questionnaires were returned due to wrong addresses. Of 5,400 eligible nurses, 2,059 nurses responded (response rate 38.1%) to the first wave. Later in the fall of 2009, invitations to join the study were sent to an additional 2,741 recently graduated nurses, of whom 905 agreed (response rate 33.0%). Together, these groups constitute SUSSH’s baseline cohort (2,964 total in the sample). At baseline 68.6% of nurses reported working in a medical hospital/outpatient clinic. Nurses also reported working in psychiatric hospital/outpatient clinic/ambulatory care (12.2%), nursing homes (8.2%), home care (7%), child health clinics (0.1%) and other nursing positions (3.9%). During both recruitment phases, invited nurses were from all over Norway, albeit with about 50% working in western Norway at the time of recruitment. Follow-up questionnaires are sent annually (with pre-paid envelopes for their return) to respondents of the first wave except for those who have died, withdrawn from the study, or have an unknown address. Each wave receives two reminders via post and are included in a lottery if they respond. Questions encompass demographics, health, work factors and lifestyles, but vary from year to year. In wave 10 of SUSSH (2018), 1,698 nurses responded to the questionnaire (61.2% response rate of baseline sample). We excluded 363 nurses who did not indicate they were currently working as nurses at the time of the survey, leaving an analytical sample of 1,335 nurses.

### Independent variables

Demographic variables included sex (“female”, “male”), age (continuous variable), children living at home (“yes”, “no”) and average hours worked per week (continuous). Background variables were workplace (“somatic hospital/department/outpatient clinic”, “psychiatric hospital/department/outpatient clinic/ambulance services”, “nursing home”, “home nursing”, “public health clinic”, “other nursing position”), percentage of full-time position at primary employer (“<50%”, “50–75%”, “76–90%”, “>90%”) and work schedule type (“day only”, “evening only” “both day and evening”, “night only”, “3-shift rotation”, “another schedule including night work”). Data were collected on self-reported fatigue, pain, sleep duration, insomnia, sleepiness, anxiety, depression, shiftwork, SWD, number of nights worked in the last year, and number of quick returns worked in the last year. Several variables were treated as both continuous and categorical depending on the analysis. Categories for variables not detailed below included shiftwork (“no”, “yes”), nights worked in the last year (“0”, “1–20”, “>20”), and number of quick returns worked last year (“0”, “1–20”, “>20”).

### Instruments

Pain was measured using six questions related to musculoskeletal complaints in various body regions within the past month (neck/shoulder/upper back, lower back, arm/wrist/hand, hip/leg/knee/foot, head and stomach) as developed by Steingrimsdottir et al. [47]. For each body region, for example “pain in the neck, shoulder or upper back”, participants indicated their pain on a four-point scale for intensity (0 = “not troubled”, 1 = “a little troubled”, 2 = “quite troubled”, 3 = “seriously troubled”), and a four-point scale for duration (1 = “1–5 days”, 2 = “6–10 days”, 3 = “11–14 days”, 4 = “15–28 days”). A complaint-severity score for each body area was constructed by multiplying the intensity score by the duration score (range 0–12). Further, a musculoskeletal complaint-severity score (MSI) was computed as the mean of all complaint-severity scores (range 0–12). Higher scores indicate a higher burden of pain. Dichotomous variables for pain by body region and MSI (“no”, “yes”) were created for chi-square analyses.

Sleep duration was categorized as “<6h” (short sleep), “6–8h” (normal sleep), and “>8h” (long sleep). Sleep duration <6h has been associated with morbidity [48] and mortality [49]. A panel of experts convened by The National Sleep Foundation considered less than 6h of sleep not recommended for adults but indicated that 6h may be appropriate [50]. We categorized normal sleep as 6-8h, this is in line with categorization used in the sleep health questionnaire RU_SATED designed by Daniel Buysse [51]. Long sleep was therefore categorized as >8h.

The Bergen Insomnia Scale [52] was used to measure insomnia symptoms. In the present study, we used an updated version of the Bergen Insomnia Scale based on inclusion criteria for insomnia disorder found in the fifth edition of the Diagnostic and Statistical Manual of Mental Disorders [53]. The Bergen Insomnia Scale records the number of days per week over the last three months participants experienced sleep-related problems and daytime consequences. Higher scores on the Bergen Insomnia Scale indicate a higher burden of insomnia symptoms. Four items relate to nocturnal problems and two to daytime consequences (6 items total). If a participant reported experiencing at least one of the nocturnal problems in the first three items ≥3 days per week and at least one daytime consequence ≥3 days per week, insomnia was coded 1 (“yes”). Cronbach’s alpha for the Bergen Insomnia Scale in this study was .83 (S5 File).

SWD was assessed using three questions based on the International Classification of Sleep Disorders (ICSD-3) [54] criteria for SWD: 1) “Do you have a work schedule that sometimes overlaps with the time you usually sleep?” 2) “If yes, does this cause insomnia and/or excessive sleepiness due to a reduced amount of sleep?” 3) “If yes, has this lasted for at least three months?” If participants answered “yes” to all three questions, SWD was coded “yes” otherwise SWD was coded “no”. Although this method of classifying SWD is a proxy and has not been validated against clinical diagnosis, it has been used in past research [55].

Daytime sleepiness was assessed with the Epworth Sleepiness Scale [56], an 8 item standardized subjective measure validated for use in Norwegian [57]. Participants were asked to estimate how likely they were to doze off in eight different situations using a 4-point scale ranging from 0 = “no risk of dozing off” to 3 = “high risk of dozing off”. Each item score is added, resulting in a composite score ranging from 0 to 24. Higher scores indicate higher daytime sleepiness and a score of 11 or higher suggests excessive sleepiness. A dichotomous variable for excessive sleepiness (“no”, “yes”) was created using this cut-off. Cronbach’s alpha for the Epworth Sleepiness Scale in the present study was .77 (S5 File).

Symptoms of anxiety and depression were assessed with a validated Norwegian version [58] of the Hospital Anxiety and Depression Scale [59]. The Hospital Anxiety and Depression Scale measures 7 non-vegetative anxiety symptoms and 7 non-vegetative depression symptoms, amounting to a total of 14 items. Participants were asked to score each item 0–3, with 0 indicating a low burden of anxiety or depressive symptoms and 3 indicating a high burden. Item scores for each subscale are added to create separate sum scores for anxiety and depression, where higher scores indicate higher burdens of anxious or depressive symptoms. A cut-off of 8 or above on each subscale score was used to categorize anxiety and depression (“no”, “yes”), respectively. This is in accordance with previous studies [58]. In the present study, Cronbach’s alphas were .84 for anxiety and .82 for depression, respectively (S5 File).

### Dependent variable

The Norwegian version [60] of the Chalder Fatigue Questionnaire (Chalder Fatigue Scale) [61], an 11 item questionnaire evaluating physical and mental fatigue, was used to assess fatigue. In order to differentiate between cases/non-cases of excessive fatigue, separate physical and mental fatigue scores were not used, but rather a global binary score [62]. Questions on fatigue are scored with a 4-point Likert scale where higher numbers indicate a higher burden of fatigue (total score range 0–33). In line with Chalder Fatigue Questionnaire guidelines [62] and other studies [22, 23], scores for each item were dichotomized (0,0,1,1) so that options such as “less than usual” or “not more than usual” = 0 and “more than usual” or “much more than usual” = 1. As per Chalder Fatigue Questionnaire guidelines, dichotomized scores were summed and scores ≥4 were categorized as excessive fatigue [62]. In the present study, the Cronbach’s alpha was .91 (S5 File) for the Chalder Fatigue Questionnaire.

### Analysis

We compared the prevalence of excessive fatigue in participants by categories of the predictors using Pearson’s chi-square tests. Continuous variables in the chi-square analysis were categorized dichotomously (“no”, “yes”) to make it possible to compare the prevalence of the outcomes in different groups.

Logistic regression analysis was used to estimate the odds ratios (OR) with 95% confidence intervals (CI) for the association between excessive fatigue and each predictor. Predictor variables were explored continuously wherever possible in logistic regressions, as continuous variables are preferred to retain as much information and statistical power as possible [63]. Categorical versions of variables were also analyzed and reported in all but the final model, as categorical variables are commonly reported and lend themselves to straightforward interpretation [63]. For continuous variables, the OR indicates the change in odds for each unit increase in the continuous variable. ORs for those reporting more severe symptoms, for example, may be considerably higher than the OR appears as reported.

We first ran crude analyses to estimate the association between excessive fatigue and each predictor individually. We then ran a model (Model 1) adjusting for age, sex, children at home and average hours worked per week for each predictor variable. We then retained the continuous versions of the variables for the final model (Model 2), except for sleep duration which remained categorized. SWD was not included in Model 2 because SWD’s criteria include insomnia and excessive sleepiness, two variables that were already included in the analysis. Additionally, MSI comprised all pain variables, and therefore could not be analyzed simultaneously with pain variables by body region in Model 2. MSI was therefore analyzed in a separate regression model that excluded the pain variables by body region but was otherwise identical. In Model 2 and the separate model for MSI, all variables were adjusted for each other as well as the demographic variables. The variables in Model 2 included: demographics (age, sex, children at home, average hours worked per week), pain (either by body region or MSI), sleep duration, insomnia, sleepiness, anxiety, depression, number of nights worked in the past year and number of quick returns in the past year.

Logistic regression analyses were checked for significance and goodness of fit, Nagelkerke R square was calculated, and outliers were retained (S9–S11 Files). Multicollinearity was assessed via variance inflation factor (VIF), no multicollinearity was found (S4 File). Model 2’s linearity of continuous variables with respect to the logit of the dependent variable was assessed (S1 and S3 Files). All statistical analyses were conducted in SPSS version 28 and Stata version 17. The threshold for statistical significance was set at p<0.05.

### Ethics

The Regional Committee for Medical Research Ethics in Western Norway (No. 088.88) approved this study. Written informed consent was obtained for each participant.

## Results

Nurses were between 31 and 69 years, mean age was 41.6 (SD = 8.2) years. The study sample included 1,202 women (90.4%) and 128 men (9.6%) (5 nurses had missing data on sex). This cohort worked on average 34.1 (SD = 6.2) hours per week. Descriptive statistics are presented in Table 1.

**Table 1:** Demographics and work-related variables among 1335 Norwegian nurses participating in the survey of shift work, sleep and health wave 10 (2018).

	Total	Number (Percent)	Mean (SD)
**Age**	1332		41.6 (8.2)
30–39 years		625 (46.9%)	
40–49 years		474 (35.6%)	
50–59 years		189 (14.2%)	
60–69 years		44 (3.3%)	
**Sex**	1330		
Female		1202 (90.4%)	
Male		128 (9.6%)	
**Children at home**	1266		
No children		383 (30.3%)	
Children		883 (69.7%)	
**Workplace**	1265		
Somatic hospital/department/outpatient clinic		683 (54.0%)	
Psychiatric Hospital/dept/outpatient clinic/ambulance		159 (12.6%)	
Nursing home		140 (11.1%)	
Home nursing		104 (8.2%)	
Public health clinic		53 (4.2%)	
Other nursing position		126 (10.0%)	
**Percentage of full-time position at primary employer**	1313		
<50%		31 (2.4%)	
50–75%		207 (15.8%)	
76–90%		260 (19.8%)	
>90%		815 (62.1%)	
**Average working hours per week**	1312		34.1 (6.2)
≤ 20 hours		67 (5.1%)	
20.1–30 hours		274 (20.9%)	
30.1–37.5 hours		757 (57.7%)	
37.6–40 hours		151 (11.5%)	
> 40 hours		63 (4.8%)	
**Work schedule type**	1308		
Day only		377 (28.8%)	
Evening only		0 (0%)	
Both day and evening		405 (31.0%)	
Night only		87 (6.7%)	
3-shift rotation		383 (29.3%)	
Another schedule including night work		56 (4.3%)	
**Shift work**	1252		
No shift work^1^		377 (30.1%)	
Shift work		875 (69.9%)	
**Night work in the last year**	1317		19.8 (35.9)
0 Nights		667 (50.6%)	
1–20		279 (21.2%)	
> 20		371 (28.2%)	
**Quick Returns**^2^ **in the last year**	1306		28.2 (37.6)
0 Quick returns		407 (31.2%)	
1–20		334 (25.6%)	
>20		565 (43.3%)	

^1^ No shift work is defined as only working days.

^2^ Less than 11 hours between consecutive work shifts

Table 2 illustrates the nurses’ health status. In total, 35.4% of the nurses reported excessive fatigue. Percentages of nurses reporting pain by body region ranged from 27% (stomach) to 70.7% (neck/shoulder/upper back), with only 7.1% of nurses reporting no pain at all. The majority (90%) slept 6–8 hours each night, the group on average slept 6.8 (SD = 0.8) hours per night. About one third of the group (30.9%) reported insomnia, 25.6% reported excessive sleepiness, 25.2% reported anxiety, 10.7% reported depression and 33.6% were categorized as having SWD.

**Table 2:** Health of 1335 Norwegian nurses participating in the survey of shift work, sleep and health wave 10 (2018).

	Total	Number (Percent)	Mean (SD)
**Fatigue**	1319		
Fatigue score^1^		1319	13.3 (4.8)
Excessive fatigue^2^		467 (35.4%)	
No excessive fatigue		852 (64.6%)	
**Pain by body region** ^3^			
**Neck/shoulder/upper back**	1330		
Neck/shoulder/upper back pain severity		1330	2.8 (3.5)
No pain		390 (29.3%)	
Pain		940 (70.7%)	
**Lower back**	1323		
Lower back pain severity		1323	2.0 (3.1)
No pain		554 (41.9%)	
Pain		769 (58.1%)	
**Arm/wrist/hand**	1315		
Arm/wrist/hand pain severity		1315	1.2 (2.6)
No pain		892 (67.8%)	
Pain		423 (32.2%)	
**Hip/leg/knee/foot**	1316		
Hip/leg/knee/foot pain severity		1316	2.1 (3.2)
No pain		622 (47.3%)	
Pain		694 (52.7%)	
**Headache/Migraine**	1324		
Headache/Migraine severity		1324	1.3 (2.1)
No headache or migraine		623 (47.1%)	
Headache or migraine present		701 (52.9%)	
**Stomach**	1325		
Stomach pain severity		1325	0.6 (1.6)
No Stomach pain		967 (73.0%)	
Stomach pain		358 (27.0%)	
**Pain using MSI**^4^ **score**	1285		
MSI (severity for pain of any type)		1285	1.7 (1.7)
No pain^5^		91 (7.1%)	
Pain of any type		1194 (92.9%)	
**Sleep duration in hours**	1197		6.8 (0.8)
Normal 6–8h		1077 (90.0%)	
Short (<6h)		99 (8.3%)	
Long (>8h)		21(1.8%)	
**Insomnia** ^6a^	1335		
Insomnia score^6b^		1315	12.0 (8.1)
No insomnia		923 (69.1%)	
Insomnia		412 (30.9%)	
**Sleepiness** ^7^	1293		
Sleepiness score		1293	8.0 (3.9)
No excessive sleepiness		962 (74.4%)	
Excessive sleepiness		331 (25.6%)	
**Anxiety** ^8^	1319		
Anxiety score		1319	5.2 (3.8)
No anxiety		987 (74.8%)	
Anxiety		332 (25.2%)	
**Depression** ^8^	1322		
Depression score		1322	3.1 (3.1)
No depression		1181 (89.3%)	
Depression		141 (10.7%)	
**Shift work disorder** ^9^	1328		
No SWD		882 (66.4%)	
SWD		446 (33.6%)	

^1^ Chalder Fatigue scale (range 0–33).

^2^ Chalder Fatigue dichotomized sum score ≥4 considered excessive fatigue.

^3^ Pain Severity by region was scored by multiplying reported pain intensity score by duration score (range 0–12).

^4^ Musculoskeletal Complaint Severity index (MSI) calculated as the mean of all complaint-severity scores (range 0–12).

^5^ Scoring 0 on the MSI for all body regions.

^6a^ Measured with the Bergen Insomnia Scale (range 0–42).

^6b^ Bergen Insomnia Scale was categorized into having vs. not having insomina based on DSM-5 criteria (this causes the N for insomnia caseness to differ from the N for insomnia scale score).

^7^ Measured with the Epworth Sleepiness Scale (range 0–24). Scores ≥11 defined as excessive sleepiness.

^8^ Measured with the Hospital Anxiety and Depression Scale (range for each 0–21). Scores ≥8 for either anxiety subscale or depression subscale defined as having anxiety or depression respectively.

^9^ Shift work disorder, assessed with 3 questions adhering to International Classification of Sleep Disorders ed. 3.

The prevalence of excessive fatigue varied by age group in Chi-square analysis (p = 0.03) and was highest in nurses aged 50–59 years (Table 3). Prevalence of excessive fatigue was higher for nurses with pain in all pain groups, as well as nurses with sleep duration <6 hours, insomnia, excessive sleepiness, anxiety and depression. Differences between groups were statistically significant.

**Table 3:** Excessive fatigue depending on demographics, sleep, work-related variables and health issues among 1335 Norwegian nurses participating in the survey of shift work, sleep and health, wave 10 (2018).

	Total	Total for subcategories	No excessive fatigue n (%)	Excessive fatigue n (%)	Chi-square (df)^a^	p-value^b^
**Age**	1316				8.870 (3)	**.031**
30–39 years		617	399 (64.7%)	218 (35.3%)		
40–49 years		468	315 (67.3%)	153 (32.7%)		
50–59 years		188	106 (56.4%)	82 (43.6%)		
60–69 years		43	32 (74.4%)	11 (25.6%)		
**Sex**	1314				0.482 (1)	0.487
Female		1187	771 (65.0%)	416 (35.0%)		
Male		127	78 (61.4%)	49 (38.6%)		
**Children at home**	1251				0.156 (1)	0.693
No children		382	250 (65.4%)	132 (34.6%)		
Children		869	557 (64.1%)	312 (35.9%)		
**Workplace**	1250				8.086 (5)	0.152
Somatic hospital/department/outpatient clinic		675	442 (65.5%)	233 (34.5%)		
Psychiatric Hospital/dept/outpatient clinic/ambulance		156	100 (64.1%)	56 (35.9%)		
Nursing home		139	78 (56.1%)	61 (43.9%)		
Home nursing		103	62 (60.2%)	41 (39.8%)		
Public health clinic		53	40 (75.5%)	13 (24.5%)		
Other nursing position		124	80 (64.5%)	44 (35.5%)		
**Percentage of full-time position at primary employer**	1297				0.268 (3)	0.966
<50%		30	19 (63.3%)	11 (36.7%)		
50–75%		203	134 (66.0%)	69 (34.0%)		
76–90%		257	164 (63.8%)	93 (36.2%)		
>90%		807	521 (64.6%)	286 (35.4%)		
**Average working hours per week**	1296				7.217 (4)	0.125
≤ 20 hours		67	36 (53.7%)	31 (46.3%)		
21.1–30 hours		268	184 (68.7%)	84 (31.3%)		
31.1–37.5 hours		752	481 (64.0%)	271 (36.0%)		
37.6–40 hours		148	101 (68.2%)	47 (31.8%)		
> 40 hours		61	36 (59.0%)	25 (41.0%)		
**Work schedule type**	1293				2.100 (4)	0.717
Day only		376	250 (66.5%)	126 (33.5%)		
Evening only		0	0 (0%)	0 (0%)		
Both day and evening		396	252 (63.6%)	144 (36.4%)		
Night only		87	56 (64.4%)	31 (35.6%)		
3-shift rotation		379	239 (63.1%)	140 (36.9%)		
Another schedule including night work		55	39 (70.9%)	16 (29.1%)		
**Nights worked the last year**	1301				0.126 (2)	0.939
0 Nights		660	424 (64.2%)	236 (35.8%)		
Nights 1–20		275	180 (65.5%)	95 (34.5%)		
Nights > 20		366	236 (64.5%)	130 (35.5%)		
**Quick Returns**^1^ **worked the last year**	1290				0.822 (2)	0.663
Quick returns 0		404	267 (66.1%)	137 (33.9%)		
Quick returns 1–20		328	212 (64.6%)	116 (35.4%)		
Quick returns >20		558	353 (63.3%)	205 (36.7%)		
**Shift Work**	1238				0.922 (1)	0.337
No shift work^2^		376	250 (66.5%)	126 (33.5%)		
Shift work		862	547 (63.5%)	315 (36.5%)		
**Pain by body region**						
**Neck/shoulder/upper back**	1314				39.330 (1)	**< .001**
No pain		385	299 (77.7%)	86 (22.3%)		
Pain		929	551 (59.3%)	378 (40.7%)		
**Lower back**	1308				29.669 (1)	**< .001**
No pain		548	401 (73.2%)	147 (26.8%)		
Pain		760	444 (58.4%)	316 (41.6%)		
**Arm/wrist/hand**	1301				60.981 (1)	**< .001**
No pain		880	633 (71.9%)	247 (28.1%)		
Pain		421	209 (49.6%)	212 (50.4%)		
**Hip/leg/knee/foot**	1300				70.186 (1)	**< .001**
No pain		613	470 (76.7%)	143 (23.3%)		
Pain		687	373 (54.3%)	314 (45.7%)		
**Headache/Migraine**	1308				43.903 (1)	**< .001**
No headache or migraine		615	455 (74.0%)	160 (26.0%)		
Headache or migraine present		693	390 (56.3%)	303 (43.7%)		
**Stomach**	1310				43.684 (1)	**< .001**
No pain		957	670 (70.0%)	287 (30.0%)		
Pain		353	177 (50.1%)	176 (49.9%)		
**Pain dichotomized from MSI**^3^ **score**	1271				15.906 (1)	**< .001**
No pain^4^		91	77 (84.6%)	14 (15.4%)		
Pain		1180	747 (63.3%)	433 (36.7%)		
**Sleep duration in hours**	1183				36.138 (2)	**< .001**
Normal (6–8h)		1065	719 (67.5%)	346 (32.5%)		
Short (<6h)		97	36 (37.1%)	61 (62.9%)		
Long (>8h)		21	14 (66.7%)	7 (33.3%)		
**Insomnia** ^5^	1319				95.954 (1)	**< .001**
No insomnia		910	667 (73.3%)	243 (26.7%)		
Insomnia		409	185 (45.2%)	224 (54.8%)		
**Sleepiness** ^6^	1281				57.252 (1)	**< .001**
No excessive sleepiness		953	673 (70.6%)	280 (29.4%)		
Excessive sleepiness		328	155 (47.3%)	173 (52.7%)		
**Anxiety** ^7^	1305				146.779 (1)	**< .001**
No anxiety		977	721 (73.8%)	256 (26.2%)		
Anxiety		328	120 (36.6%)	208 (63.4%)		
**Depression** ^7^	1308				121.308 (1)	**< .001**
No depression		1170	814 (69.6%)	356 (30.4%)		
Depression		138	30 (21.7%)	108 (78.3%)		
**Shift work disorder** ^8^	1313				42.880 (1)	**< .001**
No SWD		872	616 (70.6%)	256 (29.4%)		
SWD		441	230 (52.2%)	211 (47.8%)		

Values in **bold** are statistically significant p<0.05

^a^ Degrees of freedom.

^b^ Pearson Chi-Square or Yates’ Correction for Continuity (for 2 x 2 tables).

^1^ Less than 11 hours between consecutive work shifts.

^2^ No shift work is defined as only working days.

^3^ Musculoskeletal Complaint Severity index (MSI) calculated as the mean of all complaint-severity scores (0–12).

^4^ No pain is defined as scoring a 0 on the MSI scale.

^5^ Measured with the Bergen Insomnia Scale. Insomnia caseness based on DSM-5 criteria.

^6^ Measured with the Epworth Sleepiness Scale. Scores ≥11 defined as excessive sleepiness.

^7^ Measured with the Hospital Anxiety and Depression Scale. Scores ≥8 for either anxiety subscale or depression subscale defined as having anxiety or depression respectively.

^8^ Assessed with 3 questions adhering to International Classification of Sleep Disorders ed. 3 shift work disorder diagnostic criteria.

In the crude logistic regression analyses (Table 4) pain, sleep duration <6h, insomnia (including total insomnia score), sleepiness (both excessive sleepiness and total sleepiness score), anxiety (including total anxiety score), depression (including total depression score), SWD and number of quick returns in the past year were significantly associated with excessive fatigue, whereas demographics (age, sex, having kids at home, and average hours worked per week) and the remaining work variables (shift work, working nights and categorical quick returns) were not. Pain by body region scores had ORs for excessive fatigue ranging from 1.16 for neck (95% CI 1.12–1.20) and lower back (95% CI 1.11–1.20) regions to 1.31 (95% CI 1.23–1.40) for headache/migraine. The odds ratio for excessive fatigue was 1.66 (95% CI 1.53–1.80) for the MSI score. Nurses with sleep duration <6h, compared to nurses with normal sleep (6-8h), and nurses with insomnia, compared to those without insomnia, had more than three times the odds of excessive fatigue compared to their reference group (OR = 3.52, 95% CI 2.29–5.42, and OR = 3.32, 95% CI 2.60–4.24, respectively). Notably, nurses with anxiety had 4.88 times (95% CI 3.74–6.37) and nurses with depression 8.23 times (95% CI 5.39–12.57) the odds of reporting excessive fatigue. Nurses with SWD had a more than two-fold increased odds (OR = 2.21, 95% CI 1.74–2.80) of excessive fatigue.

**Table 4:** Crude and logistic regression analyses with excessive fatigue as the dependent variable among 1335 Norwegian nurses participating in the survey of shift work, sleep and health, wave 10 (2018).

	Number	Crude Analyses		Model 1*		Model 2**	
	(Crude, Model 1, Model 2)	OR	95% CI	OR	95% CI	OR	95% CI
Demographic variables							
**Age**	1316						
Age continuous		1.00	0.99–1.02				
**Sex**	1314						
Female		1.00	(ref)				
Male		1.16	0.80–1.70				
**Kids at home**	1251						
No kids at home		1.00	(ref)				
Kids at home		1.06	0.82–1.37				
**Average hours worked**	1296						
30.1–37.5h		1.00	(ref)				
≤ 20h		1.53	0.93–2.53				
21.1 – 30h		0.81	0.60–1.09				
37.6 – 40h		0.83	0.57–1.20				
> 40h		1.23	0.72–2.10				
**Pain Severity** ^1^							
Neck/shoulder/upper back	1314, 1219, 997	**1.16**	1.12–1.20	**1.16**	1.13–1.21	0.98	0.93–1.04
Lower back	1308, 1211, 997	**1.16**	1.11–1.20	**1.15**	1.11–1.19	0.97	0.92–1.03
Arm/wrist/hand	1301, 1208, 997	**1.19**	1.14–1.25	**1.20**	1.14–1.26	**1.09** ^a^	1.02–1.17
Hip/leg/knee/foot	1300, 1207, 997	**1.19**	1.15–1.24	**1.21**	1.16–1.26	**1.11**	1.05–1.18
Headache/Migraine	1308, 1213, 997	**1.31**	1.23–1.40	**1.31**	1.23–1.40	**1.16**	1.07–1.27
Stomach	1310, 1214, 997	**1.26**	1.17–1.37	**1.25**	1.15–1.36	1.02	0.92–1.13
MSI^2^	1271, 1182, 997	**1.66**	1.53–1.80	**1.70**	1.56–1.86		
**Sleep Duration in hours**	1183, 1100, 997						
Sleep dur. (6–8h)		1.00	(ref)	1.00	(ref)	1.00	(ref)
Short sleep (<6h)		**3.52**	2.29–5.42	**3.16**	2.01–5.00	**2.02** ^b^	1.08–3.77
Long sleep (>8h+)		1.04	0.42–2.60	0.99	0.38–2.57	0.72	0.19–2.79
**Insomnia** ^3^	1319, 1222						
No insomnia		1.00	(ref)	1.00	(ref)		
Insomnia		**3.32**	2.60–4.24	**3.15**	2.44–4.07		
Insomnia score	1301, 1205, 997	**1.13**	1.11–1.15	**1.13**	1.11–1.15	**1.05**	1.03–1.08
**Sleepiness** ^4^	1281, 1188						
No excessive sleepiness		1.00	(ref)	1.00	(ref)		
Excessive sleepiness		**2.68**	2.07–3.47	**2.63**	2.01–3.45		
Sleepiness score	1281, 1188, 997	**1.19**	1.15–1.22	**1.18**	1.14–1.23	**1.11**	1.06–1.17
**Anxiety** ^5^	1305, 1209						
No Anxiety		1.00	(ref)	1.00	(ref)		
Anxiety		**4.88**	3.74–6.37	**5.00**	3.78–6.61		
Anxiety score	1305, 1209, 997	**1.27**	1.23–1.32	**1.29**	1.24–1.34	**1.09,** ^c^	1.03–1.16
**Depression** ^5^	1308, 1212						
No depression		1.00	(ref)	1.00	(ref)		
Depression		**8.23**	5.39–12.57	**8.57**	5.47–13.43		
Depression score	1308, 1212, 997	**1.42**	1.36–1.49	**1.44**	1.37–1.52	**1.24**	1.16–1.33
**Shift work**	1238, 1156						
No shift work^6^		1.00	(ref)	1.00	(ref)		
Shift work		1.14	0.89–1.48	1.18	0.90–1.56		
**SWD** ^7^	1313, 1217						
No SWD		1.00	(ref)	1.00	(ref)		
SWD		**2.21**	1.74–2.80	**2.25**	1.76–2.89		
**Night work in the last year**	1301, 1216						
Nights (ref 0 nights)		1.00	(ref)	1.00	(ref)		
Nights 1–20		0.95	0.71–1.27	0.92	0.68–1.26		
Nights > 20		0.99	0.76–1.29	0.96	0.72–1.27		
# of nights last year (cont.)	1301, 1216, 997	1.00	1.00–1.00	1.00	1.00–1.00	1.00	1.00–1.00
**Quick Returns** ^8^	1290, 1208						
Quick returns (ref 0)		1.00	(ref)	1.00	(ref)		
Quick returns 1–20		1.07	0.79–1.45	1.07	0.78–1.47		
Quick returns >20		1.13	0.87–1.48	1.16	0.87–1.55		
# of quick returns last year	1290, 1208, 997	**1.01**	1.00–1.01	**1.01**	1.00–1.01	1.00	1.00–1.01

Except where otherwise noted, ORs in **bold** font are statistically significant p<0.001

*Model 1, each variable separately adjusted for age, sex, kids at home and average hours worked.

**Model 2, All continuous variables included plus sleep duration (included categorically) and adjusted for age, sex, kids at home and average hours worked and each other. MSI not included because it is built from the pain by body region variables. MSI was run in a separate Model 2 and was still significantly associated with fatigue.

^1^Pain Severity scales multiply reported pain intensity score by duration score.

^2^ Musculoskeletal complaint severity calculated as the mean of all complaint-severity scores.

^3^ Measured with the Bergen Insomnia Scale. Insomnia caseness based on DSM-5 criteria (this causes the N for insomnia caseness to differ from the N for insomnia scale score).

^4^ Measured with the Epworth Sleepiness Scale. Scores ≥11 defined as excessive sleepiness.

^5^ Measured with the Hospital Anxiety and Depression Scale. Scores ≥8 for either anxiety subscale or depression subscale defined as having anxiety or depression respectively.

^6^ Shift work disorder. No shift work is defined as only working days.

^7^Shift work disorder assessed with 3 questions adhering to International Classification of Sleep Disorders ed. 3 shift work disorder diagnostic criteria. This variable was not included in Model 2 because it is based on insomnia and sleepiness symptoms.

^8^ Quick Returns are less than 11 hours between consecutive work shifts.

^a^ p = 0.017

^b^ p = 0.028

^c^ p = 0.002

All statistically significant findings from the crude analyses remained (pain, sleep duration <6h, insomnia, sleepiness, anxiety, depression, SWD and number of quick returns in the past year) when adjusting for age, sex, children at home, and average hours worked per week (Table 4, Model 1). When also adjusting for all continuous variables, sleep duration (categorical) and demographics, neck/shoulder/upper back, lower back and stomach pain severity scores were no longer significant, but arm/wrist/hand, hip/leg/knee/foot, headache/migraine pain severity remained statistically significant (Table 4, Model 2). Headache/migraine still had the highest OR for excessive fatigue of the pain variables in a single body region with an adjusted odds ratio (aOR) of 1.16 (95% CI 1.07–1.27). Sleep duration <6 hours (aOR 2.02 (95% CI 1.08–3.77)), insomnia score (aOR 1.05 (95% CI 1.03–1.08)), sleepiness score (aOR 1.11 (95% CI 1.06–1.17)), anxiety score (aOR 1.09 (95% CI 1.03–1.16)) and depression score (aOR 1.24 (95% CI 1.16–1.33) remained statistically significant in the final analysis, but number of quick returns in the past year was no longer significant. MSI was run in a separate logistic regression (identical to Model 2 except for the use of MSI instead of pain by body region variables) and remained statistically significant with an aOR of 1.27 (95% CI 1.13–1.42), not shown in Table 4.

## Discussion

Our study found that pain, sleep duration <6h, insomnia, sleepiness, anxiety, and depression were significantly related to excessive fatigue even after controlling for all other variables present in the model. SWD was also associated significantly with excessive fatigue. Of our hypotheses, H1 (nurses with greater pain severity will more likely have excessive fatigue), H2 (nurses with sleep problems will more likely have excessive fatigue), and H3 (nurses with high levels of anxiety and depressive symptoms will more likely have excessive fatigue) were supported. H4 (nurses with shift work, night work and quick returns will more likely have excessive fatigue), however, was unsupported based on the fully adjusted model (Model 2).

Unlike some studies [17–21], we found no significant relationship between excessive fatigue and female sex. However, our findings correspond with previous studies in our SUSSH cohort where sex was not significantly associated with excessive fatigue [22, 23]. Our analyses found no significant relationship between excessive fatigue and age, which corroborates some [20, 22, 23] of the conflicting previous research. The current study supports the relationships between excessive fatigue and pain [30, 31], short sleep duration [33], insomnia [35] and anxiety and depression [38, 39].

We had no a priori hypotheses of which body regions may associate or not associate with fatigue. Because the measure we used offered both regional as well as global scores for pain we felt that both warranted investigation. Our results indicate the importance of pain in in relation to excessive fatigue, even in a presumably healthy cohort. The exact implications of the results of pain by body region must be interpreted with caution, however. That neck/shoulder/upper back, lower back and stomach pain were no longer significant in Model 2, but arm/wrist/hand, hip/leg/knee/foot, headache/migraine pain remained significant may be due to loss of power due to the study sample decreasing when all variables were included, or to potential/real differences in how pain in those regions affects a nurse’s fatigue.

Some of the results were imprecise with broad confidence intervals due to few nurses within each category of predictor and outcome–these results should be interpreted with caution. Our category for long sleep duration (>8h), for example, included a very small number of nurses reporting sleep >8h (n = 21) which affected the statistical power. Additionally, a healthy worker effect [64] may also be present, as long sleep is often associated with poor health [65]. Our findings on sleepiness corroborate Farag et al.’s study wherein fatigue was significantly associated with increased daytime sleepiness [17].

We found no significant relationship between excessive fatigue and several shift work-related factors, unlike other studies from Taiwan [43] and Sweden [21]. This contrasting finding may potentially be explained by the fact that the SUSSH nurses had already been followed for 10 years in 2018, and nurses who tolerated shift work poorly may have changed work schedules or careers by this point. In contrast to a previous study [40], we found no significant relationship between excessive fatigue and working nights in our group. This may be explained by operationalization differences. Åkerstedt et al. focused on work-related fatigue assessed with the question “do you feel that your work schedule causes fatigue (yes/no)” [40]. Excessive fatigue as measured in our cohort was not specific to the work schedule and therefore may have missed a relationship between work schedule-related fatigue and night work. Additionally a study by Øyane et al. found current night work to be associated with chronic fatigue [42]. However, that this latter study found no association between cumulative nights worked within the last year and chronic fatigue [42] is in line with our findings. Additionally, we expected that age would be an important confounder in the relationships between excessive fatigue and nights or quick returns, but we did not see evidence of this in the present study.

The present results corroborate a previous study in our cohort of nurses wherein number of quick returns in the past year was associated with excessive fatigue in crude, but not adjusted analyses [22]. However, these findings contradict other past studies [23, 40]. That Åkerstedt et al. [40] found a significant relationship between quick returns and fatigue in adjusted analyses may be due to the different operationalization of fatigue discussed with regard to night work, or other methodological differences. For example, Åkerstedt et al. adjusted analyses for age, gender, socioeconomics, and physically intensive work [40] but not for pain, sleep, mental-health or work schedule factors. Flo et al. [23] also used SUSSH data (from 2009 and 2010) and found that the number of quick returns in 2009 predicted pathological fatigue (operationalized in the same manner as excessive fatigue in our study) in 2010, and a reduction in quick returns from 2009 to 2010 was protective against pathological fatigue in 2010 [23]. It may be that those in our cohort who tolerated quick returns were more likely to continue working and therefore were overrepresented after nearly a decade of follow-up. Alternately, the cross-sectional nature of the present study may have missed a possible longitudinal relationship. Further longitudinal studies are needed to elucidate the relationship between fatigue and quick returns.

The current study found a significant association between excessive fatigue and SWD in both crude analyses and analyses adjusted for demographics. This is in line with a study from the first SUSSH wave (2008/2009) [37]. Our study did not include SWD in the final model (Model 2), because insomnia and excessive sleepiness, two variables included in the final model, are included in the criteria for SWD (participants were asked if their work schedule overlapped with normal sleep hours and if that caused insomnia and/or excessive sleepiness due to reduced amount of sleep). Therefore, associations between SWD and the other variables could not be explored in the fully adjusted model.

### Limitations and strengths

As the present study relies on self-reported data, there is a risk of inaccurate recall. The cross-sectional nature of the study negates the ability to interpret causality, and the cohort of nurses containing only ~10% men limits generalizability to other occupations and to male populations. Additional variables that may associate with fatigue (for example stress, burnout, nutrition, and diseases) have not been considered in our analyses. Due to the lack of clinical interviews, sleep diaries, etc., no formal diagnoses of participants could be made. Since all measures were based on self-report and collected at the same time point, there is a risk for the results to be influenced by the common method bias.

After initial SUSSH recruitment, attrition has been low. The present study had 61.2% response rate from baseline participation, within ~60% ±20%, the amount found as a possible norm for general population response rates in an investigation of 175 studies by Yehuda Baruch [66]. Conversely, the initial response rate in the SUSSH cohort at baseline was quite low (38.1%), which could possibly indicate selection bias and negatively impact the generalizability of the study. Nonetheless, as nonparticipation is often associated with poorer health [67], it is unlikely that the prevalence of excessive fatigue in the cohort was inflated due to differences between responders and non-responders. If anything, a healthy worker effect [64] may be present. Non-responders may have developed severe fatigue and therefore changed workplace, work schedule or quit nursing, diminishing associations between excessive fatigue and the variables in question. Yet, we still found significant associations between excessive fatigue and pain, sleep, and mental health.

SUSSH’s large and homogenous cohort limits confounding by factors such as education level, income, work schedule and workload. Additionally, it reflects the ~90% female sex of the Norwegian nursing population at large [68]. The scales we used to measure fatigue [22, 23], pain [47], insomnia [52], sleepiness [57], depression and anxiety [58] and SWD [55] are well-established or in line with past epidemiological studies.

A sufficiently large sample size may offer statistically significant results for relatively small and potentially meaningless differences [69]. Our sample is relatively large, therefore there is a risk of statistically significant findings for non-clinical/practical differences between those reporting and not reporting excessive fatigue. In other words, results which fail to be clinically significant. However, the sizable significant ORs for independent variables that were categorized or dichotomized range in the adjusted analyses from over 2x to over 8x the odds of excessive fatigue (OR 2.25 (95% CI 1.76–2.89) for SWD–OR 8.57 (95% CI 5.47–13.43) for depression) which is above the threshold that seems to reflect practical significance [70]. Further, chi-square tables (for example indicating that 62.9% of nurses with sleep <6h, 63.4% with anxiety and 78.3% with depression have excessive fatigue) visibly demonstrate results that are meaningfully and clinically different.

The ORs for the associations appear small for several variables. However, as mentioned in the analysis section, in the case of continuous variables, the odds ratio indicates the change in odds for each unit of change in the continuous variable. Therefore, the lowest OR in our final model, aOR 1.09 (95% CI 1.02–1.17) for arm/wrist/hand pain, translates to a 9% increase of odds of reporting excessive fatigue for each unit increase on the arm/wrist/hand pain score (the same is true for anxiety aOR 1.09 (95% CI 1.03–1.16)). Whereas the highest ORs, MSI (aOR 1.27 (95% CI 1.13–1.42)) and depression ((aOR 1.24 (95% CI 1.16–1.33)) translate to a 27% and 24% increase in odds of reporting excessive fatigue for each unit increase on their respective scales.

Other research has investigated associations between fatigue and pain, sleep, work and mental health variables. This study is the first, to our knowledge, to examine all these variables together and thereby demonstrate that relationships between fatigue and pain, sleep, mental health, and work factors exist independently of each other. Further, this study is among a small number of others [31, 32] examining pain and fatigue in the context of a working, presumably healthy cohort.

## Conclusions

Pain by body area as well as MSI, sleep duration <6h, insomnia, sleepiness, anxiety, depression, SWD and number of quick returns in the past year were associated with having excessive fatigue. Similar findings were seen after controlling for age, sex, children at home and average hours worked. Associations were found between excessive fatigue and pain severity (arm/wrist/hand, hip/leg/knee/foot, and headache/migraine), sleep duration <6h, insomnia, sleepiness, anxiety and depression in the final fully adjusted model (Model 2), where all variables plus demographics were included.

Future research should include objective/clinical measures of fatigue, pain, sleep, mental health and work factors from objective recordings as well as health and employee registries. Longitudinal studies which can elucidate the temporal relationship between the variables in question will also help advance the field. Studies on quick returns and night work in seasoned versus inexperienced nurses may help illuminate the association or lack of association between excessive fatigue and these factors. Other factors that may be associated with fatigue should be explored, including stress, burnout, nutrition, and diseases. Further research is also necessary on what factors lead to chronically fatigued nurses, and on workplace strategies to reduce excessive fatigue in nurses and prevent nurse absence and turnover. Priorities should include interventions for nurses with headaches/migraines, high MSI pain severity, sleep duration <6 hours, insomnia, sleepiness, anxiety, depression and SWD. Schedule changes and other low-risk interventions (such as mediation for pain) may be explored for efficacy against excessive fatigue.

## Supporting information

S1 File Box-Tidwell test. (SPV)

S2 File Check of variables that comprise scales. (SPV)

S3 File Histograms and linearity checks using stata. (DOCX)

S4 File Multicollinearity check. (SPV)

S5 File Reliability analyses. (SPV)

S6 File Output for Table 1. (SPV)

S7 File Output for Table 2. (SPV)

S8 File Output for Table 3. (SPV)

S9 File Output for crude logistic regression analyses. (SPV)

S10 File Output for Model 1 logistic regression analyses. (SPV)

S11 File Output for Model 2 logistic regression analyses. (SPV)

S12 File SUSSH questionnaire 2018. (PDF)

S13 File Syntax. (SPS)

## References

[1] Aaronson LS, Teel CS, Cassmeyer V, Neuberger GB, Pallikkathayil L, Pierce J, et al. Defining and measuring fatigue. Image: The Journal of Nursing Scholarship. 1999;31(1):45–50. 10.1111/j.1547-5069.1999.tb00420.x 10081212

[2] Pallesen S, Bjorvatn B. Workload and Fatigue. Space Safety and Human Performance 2017. p. 19–33.

[3] Cairns SP, Knicker AJ, Thompson MW, Sjøgaard G. Evaluation of models used to study neuromuscular fatigue. Exercise and sport sciences reviews. 2005;33(1):9–16. 15640715

[4] Dawson D, Noy YI, Härmä M, Åkerstedt T, Belenky G. Modelling fatigue and the use of fatigue models in work settings. Accident Analysis & Prevention. 2011;43(2):549–64. doi: 10.1016/j.aap.2009.12.030 21130216

[5] Gandevia SC. Spinal and supraspinal factors in human muscle fatigue. Physiological reviews. 2001. doi: 10.1152/physrev.2001.81.4.1725 11581501

[6] Boksem MA, Tops M. Mental fatigue: costs and benefits. Brain research reviews. 2008;59(1):125–39. doi: 10.1016/j.brainresrev.2008.07.001 18652844

[7] Techera U, Hallowell M, Stambaugh N, Littlejohn R. Causes and consequences of occupational fatigue. Journal of occupational and environmental medicine. 2016;58(10):961–73. 27525527 10.1097/JOM.0000000000000837

[8] Fukuda K, Straus SE, Hickie I, Sharpe MC, Dobbins JG, Komaroff A, et al. The chronic fatigue syndrome: a comprehensive approach to its definition and study. Annals of internal medicine. 1994;121(12):953–9. 7978722 10.7326/0003-4819-121-12-199412150-00009

[9] Loeppke R, Taitel M, Richling D, Parry T, Kessler RC, Hymel P, et al. Health and productivity as a business strategy. Journal of Occupational and Environmental Medicine. 2007:712–21. doi: 10.1097/JOM.0b013e318133a4be 17622843

[10] Ricci JA, Chee E, Lorandeau AL, Berger J. Fatigue in the US workforce: prevalence and implications for lost productive work time. Journal of Occupational and Environmental Medicine. 2007:1–10. 17215708 10.1097/01.jom.0000249782.60321.2a

[11] Galland-Decker C, Marques-Vidal P, Vollenweider P. Prevalence and factors associated with fatigue in the Lausanne middle-aged population: a population-based, cross-sectional survey. BMJ Open. 2019;9(8):e027070. doi: 10.1136/bmjopen-2018-027070 31446404 PMC6720133

[12] Cullen W, Kearney Y, Bury G. Prevalence of fatigue in general practice. Irish Journal of Medical Science. 2002;171(1):10–2. doi: 10.1007/BF03168931 11993585

[13] Krogstad H, Loge JH, Grotmol KS, Kaasa S, Kiserud CE, Salvesen Ø, et al. Symptoms in the general Norwegian adult population-prevalence and associated factors. BMC Public Health. 2020;20(1):1–12. 32576168 10.1186/s12889-020-09109-2 PMC7310321

[14] Son C-G. Review of the prevalence of chronic fatigue worldwide. The Journal of Korean Medicine. 2012;33(2):25–33.

[15] Oulton JA. The global nursing shortage: an overview of issues and actions. Policy, Politics, & Nursing Practice. 2006;7(3_suppl):34S–9S. doi: 10.1177/1527154406293968 17071693

[16] WHO. State of the world’s nursing 2020: investing in education, jobs and leadership. 2020. Report No.: 9240003274.

[17] Farag A, Scott L, Perkhounkova Y, Saeidzadeh S, Hein M. A human factors approach to evaluate predicators of acute care nurse occupational fatigue. Applied Ergonomics. 2022;100:103647. doi: 10.1016/j.apergo.2021.103647 34837749

[18] Åkerstedt T, Knutsson A, Westerholm P, Theorell T, Alfredsson L, Kecklund G. Mental fatigue, work and sleep. Journal of Psychosomatic Research. 2004;57(5):427–33. doi: 10.1016/j.jpsychores.2003.12.001 15581645

[19] Engberg I, Segerstedt J, Waller G, Wennberg P, Eliasson M. Fatigue in the general population-associations to age, sex, socioeconomic status, physical activity, sitting time and self-rated health: the northern Sweden MONICA study 2014. BMC Public Health. 2017;17(1):1–9. 28806984 10.1186/s12889-017-4623-y PMC5557471

[20] Hinz A, Weis J, Brähler E, Mehnert A. Fatigue in the general population: German normative values of the EORTC QLQ-FA12. Quality of Life Research. 2018;27(10):2681–9. doi: 10.1007/s11136-018-1918-0 29909484

[21] Tucker P, Leineweber C, Kecklund G. Comparing the acute effects of shiftwork on mothers and fathers. Occupational Medicine. 2021;71(9):414–21. doi: 10.1093/occmed/kqab083 34165560 PMC8703007

[22] Eldevik MF, Flo E, Moen BE, Pallesen S, Bjorvatn B. Insomnia, excessive sleepiness, excessive fatigue, anxiety, depression and shift work disorder in nurses having less than 11 hours in-between shifts. PLoS One. 2013;8(8):e70882. doi: 10.1371/journal.pone.0070882 23976964 PMC3744484

[23] Flo E, Pallesen S, Moen BE, Waage S, Bjorvatn B. Short rest periods between work shifts predict sleep and health problems in nurses at 1-year follow-up. Occupational and Environmental Medicine. 2014;71(8):555–61. doi: 10.1136/oemed-2013-102007 24919881

[24] Schwarz R, Krauss O, Hinz A. Fatigue in the general population. Oncology Research and Treatment. 2003;26(2):140–4. doi: 10.1159/000069834 12771522

[25] Bjorvatn B, Dale S, Hogstad‐Erikstein R, Fiske E, Pallesen S, Waage S. Self‐reported sleep and health among Norwegian hospital nurses in intensive care units. Nursing in Critical Care. 2012;17(4):180–8. doi: 10.1111/j.1478-5153.2012.00504.x 22698160

[26] Watt T, Groenvold M, Bjorner JB, Noerholm V, Rasmussen N-A, Bech P. Fatigue in the Danish general population. Influence of sociodemographic factors and disease. Journal of Epidemiology & Community Health. 2000;54(11):827–33. Erratum in: Journal of Epidemiology & Community Health. 2001;55(3):216. doi: 10.1136/jech.54.11.827 11027196 PMC1731588

[27] Jump RL, Robinson ME, Armstrong AE, Barnes EV, Kilbourn KM, Richards HB. Fatigue in systemic lupus erythematosus: contributions of disease activity, pain, depression, and perceived social support. The Journal of Rheumatology. 2005;32(9):1699–705. 16142863

[28] Hwang SS, Chang VT, Rue M, Kasimis B. Multidimensional independent predictors of cancer-related fatigue. Journal of Pain and Symptom Management. 2003;26(1):604–14. doi: 10.1016/s0885-3924(03)00218-5 12850643

[29] Huyser BA, Parker JC, Thoreson R, Smarr KL, Johnson JC, Hoffman R. Predictors of subjective fatigue among individuals with rheumatoid arthritis. Arthritis & Rheumatism. 1998;41(12):2230–7. doi: 10.1002/1529-0131(199812)41:12&lt;2230::AID-ART19&gt;3.0.CO;2-D 9870880

[30] Creavin ST, Dunn KM, Mallen CD, Nijrolder I, van der Windt DA. Co-occurrence and associations of pain and fatigue in a community sample of Dutch adults. European Journal of Pain. 2010;14(3):327–34. doi: 10.1016/j.ejpain.2009.05.010 19540139

[31] Daneshmandi H, Choobineh A, Ghaem H, Alhamd M, Fakherpour A. The effect of musculoskeletal problems on fatigue and productivity of office personnel: a cross-sectional study. Journal of Preventive Medicine and Hygiene. 2017;58(3):E252. 29123372 PMC5668935

[32] Silva TPDd, Araújo WNd, Stival MM, Toledo AMd, Burke TN, Carregaro RL. Musculoskeletal discomfort, work ability and fatigue in nursing professionals working in a hospital environment. Revista da Escola de Enfermagem da USP. 2018;52. 10.1590/S1980-220X2017022903332 29898170

[33] Pilcher JJ, Walters AS. How sleep deprivation affects psychological variables related to college students’ cognitive performance. Journal of American College Health. 1997;46(3):121–6. doi: 10.1080/07448489709595597 9394089

[34] Boolani A, O’Connor PJ, Reid J, Ma S, Mondal S. Predictors of feelings of energy differ from predictors of fatigue. Fatigue: Biomedicine, Health & Behavior. 2019;7(1):12–28.

[35] Lichstein KL, Means MK, Noe SL, Aguillard R. Fatigue and sleep disorders. Behaviour Research and Therapy. 1997;35(8):733–40. doi: 10.1016/s0005-7967(97)00029-6 9256516

[36] Hossain JL, Ahmad P, Reinish LW, Kayumov L, Hossain NK, Shapiro CM. Subjective fatigue and subjective sleepiness: two independent consequences of sleep disorders? Journal of Sleep Research. 2005;14(3):245–53. doi: 10.1111/j.1365-2869.2005.00466.x 16120099

[37] Flo E, Pallesen S, Magerøy N, Moen BE, Grønli J, Hilde Nordhus I, et al. Shift work disorder in nurses–assessment, prevalence and related health problems. PLoS One. 2012;7(4):e33981. doi: 10.1371/journal.pone.0033981 22485153 PMC3317447

[38] Demyttenaere K, De Fruyt J, Stahl SM. The many faces of fatigue in major depressive disorder. International Journal of Neuropsychopharmacology. 2005;8(1):93–105. doi: 10.1017/S1461145704004729 15482632

[39] Vassend O, Røysamb E, Nielsen CS, Czajkowski NO. Fatigue symptoms in relation to neuroticism, anxiety-depression, and musculoskeletal pain. A longitudinal twin study. PLoS One. 2018;13(6):e0198594. doi: 10.1371/journal.pone.0198594 29879175 PMC5991664

[40] Åkerstedt T, Kecklund G. What work schedule characteristics constitute a problem to the individual? A representative study of Swedish shift workers. Applied Ergonomics. 2017;59:320–5. doi: 10.1016/j.apergo.2016.09.007 27890143

[41] Tucker P, Brown M, Dahlgren A, Davies G, Ebden P, Folkard S, et al. The impact of junior doctors’ worktime arrangements on their fatigue and well-being. Scandinavian Journal of Work, Environment & Health. 2010:458–65. doi: 10.5271/sjweh.2985 20414629

[42] Øyane NM, Pallesen S, Moen BE, Åkerstedt T, Bjorvatn B. Associations between night work and anxiety, depression, insomnia, sleepiness and fatigue in a sample of Norwegian nurses. PLoS One. 2013;8(8):e70228. doi: 10.1371/journal.pone.0070228 23950914 PMC3737208

[43] Yuan SC, Chou MC, Chen CJ, Lin YJ, CHEN MC, LIU HH, et al. Influences of shift work on fatigue among nurses. Journal of Nursing Management. 2011;19(3):339–45. doi: 10.1111/j.1365-2834.2010.01173.x 21507104

[44] Katsifaraki M, Nilsen KB, Wærsted M, Knardahl S, Lie J-AS, Bjorvatn B, et al. The association of sleepiness, insomnia, sleep disturbance and pain: a study amongst shiftworking nurses. Sleep and Biological Rhythms. 2018;16(1):133–40.

[45] Matre D, Nilsen KB, Katsifaraki M, Waage S, Pallesen S, Bjorvatn B. Pain complaints are associated with quick returns and insomnia among Norwegian nurses, but do not differ between shift workers and day only workers. International Archives of Occupational and Environmental Health. 2020;93(3):291–9. doi: 10.1007/s00420-019-01481-w 31691014

[46] Snekkevik H, Eriksen HR, Tangen T, Chalder T, Reme SE. Fatigue and depression in sick-listed chronic low back pain patients. Pain Medicine. 2014;15(7):1163–70. doi: 10.1111/pme.12435 24716799 PMC4265279

[47] Steingrımsdóttir OA, Vøllestad NK, Røe C, Knardahl S. Variation in reporting of pain and other subjective health complaints in a working population and limitations of single sample measurements. Pain. 2004;110(1–2):130–9. doi: 10.1016/j.pain.2004.03.016 15275760

[48] Cappuccio FP, Miller MA. Sleep and cardio-metabolic disease. Current cardiology reports. 2017;19(11):1–9. doi: 10.1007/s11886-017-0916-0 28929340 PMC5605599

[49] Fernandez-Mendoza J, He F, Calhoun SL, Vgontzas AN, Liao D, Bixler EO. Objective short sleep duration increases the risk of all-cause mortality associated with possible vascular cognitive impairment. Sleep Health. 2020;6(1):71–8. doi: 10.1016/j.sleh.2019.09.003 31759934 PMC6995415

[50] Hirshkowitz M, Whiton K, Albert SM, Alessi C, Bruni O, DonCarlos L, et al. National Sleep Foundation’s sleep time duration recommendations: methodology and results summary. Sleep health. 2015;1(1):40–3. doi: 10.1016/j.sleh.2014.12.010 29073412

[51] Buysse DJ. Sleep health: can we define it? Does it matter? Sleep. 2014;37(1):9–17. doi: 10.5665/sleep.3298 24470692 PMC3902880

[52] Pallesen S, Bjorvatn B, Nordhus IH, Sivertsen B, Hjørnevik M, Morin CM. A new scale for measuring insomnia: the Bergen Insomnia Scale. Perceptual and motor skills. 2008;107(3):691–706. doi: 10.2466/pms.107.3.691-706 19235401

[53] APA. Diagnostic and statistical manual of mental disorders: American Psychiatric Association; 2013. 591–643 p.

[54] AASM. The International Classification of Sleep Disorders (ICSD-3): American Academy of Sleep Medicine; 2014.

[55] Blytt KM, Bjorvatn B, Moen BE, Pallesen S, Harris A, Waage S. The association between shift work disorder and turnover intention among nurses. BMC Nursing. 2022;21(1):1–8. 35668393 10.1186/s12912-022-00928-9 PMC9169346

[56] Johns MW. A new method for measuring daytime sleepiness: the Epworth sleepiness scale. Sleep. 1991;14(6):540–5. doi: 10.1093/sleep/14.6.540 1798888

[57] Pallesen S, Nordhus IH, Omvik S, Sivertsen B, Tell GS, Bjorvatn B. Prevalence and risk factors of subjective sleepiness in the general adult population. Sleep. 2007;30(5):619–24. doi: 10.1093/sleep/30.5.619 17552377

[58] Bjelland I, Dahl AA, Haug TT, Neckelmann D. The validity of the Hospital Anxiety and Depression Scale: an updated literature review. Journal of Psychosomatic Research. 2002;52(2):69–77. 11832252 10.1016/s0022-3999(01)00296-3

[59] Zigmond AS, Snaith RP. The hospital anxiety and depression scale. Acta Psychiatrica Scandinavica. 1983;67(6):361–70. doi: 10.1111/j.1600-0447.1983.tb09716.x 6880820

[60] Loge JH, Ekeberg Ø, Kaasa S. Fatigue in the general Norwegian population: normative data and associations. Journal of Psychosomatic Research. 1998;45(1):53–65. doi: 10.1016/s0022-3999(97)00291-2 9720855

[61] Chalder T, Berelowitz G, Pawlikowska T, Watts L, Wessely S, Wright D, et al. Development of a fatigue scale. Journal of Psychosomatic Research. 1993;37(2):147–53. doi: 10.1016/0022-3999(93)90081-p 8463991

[62] Jackson C. The Chalder Fatigue Scale (CFQ 11). Occupational Medicine. 2014;65(1):86–. 10.1093/occmed/kqu168 25559796

[63] Altman DG, Royston P. The cost of dichotomising continuous variables. Bmj. 2006;332(7549):1080. doi: 10.1136/bmj.332.7549.1080 16675816 PMC1458573

[64] Chowdhury R, Shah D, Payal AR. Healthy worker effect phenomenon: revisited with emphasis on statistical methods–a review. Indian Journal of Occupational and Environmental Medicine. 2017;21(1):2. doi: 10.4103/ijoem.IJOEM_53_16 29391741 PMC5763838

[65] Wang S, Wu Y, Ungvari GS, Ng CH, Forester BP, Gatchel JR, et al. Sleep duration and its association with demographics, lifestyle factors, poor mental health and chronic diseases in older Chinese adults. Psychiatry Research. 2017;257:212–8. doi: 10.1016/j.psychres.2017.07.036 28777953

[66] Baruch Y. Response rate in academic studies-A comparative analysis. Human Relations. 1999;52(4):421–38.

[67] Knudsen AK, Hotopf M, Skogen JC, Øverland S, Mykletun A. The health status of nonparticipants in a population-based health study: the Hordaland Health Study. American Journal of Epidemiology. 2010;172(11):1306–14. doi: 10.1093/aje/kwq257 20843863

[68] Køber T, Vigran Å. Stort omfang av deltidsarbeid. 2011. Report No.: 0809–4713.

[69] Sullivan GM, Feinn R. Using Effect Size—or Why the P Value Is Not Enough. Journal of Graduate Medical Education. 2012;4(3):279–82. doi: 10.4300/JGME-D-12-00156.1 23997866 PMC3444174

[70] Ferguson CJ. An effect size primer: a guide for clinicians and researchers. 2016.

