# Supplementary material for: Associations between excessive fatigue and pain, sleep, mental-health and work factors in Norwegian nurses
Source: PLoS One. 2023 Apr 4;18(4):e0282734. doi: 10.1371/journal.pone.0282734 (PMC10072460; doi:10.1371/journal.pone.0282734)

# Histograms and linearity checks using Stata

# Histograms

## histogram alder_2018 age


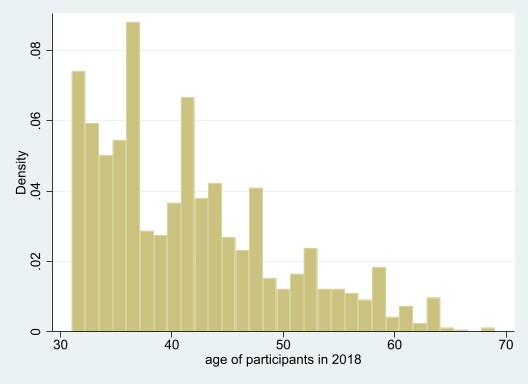


## histogram sleep_dur Sleep duration


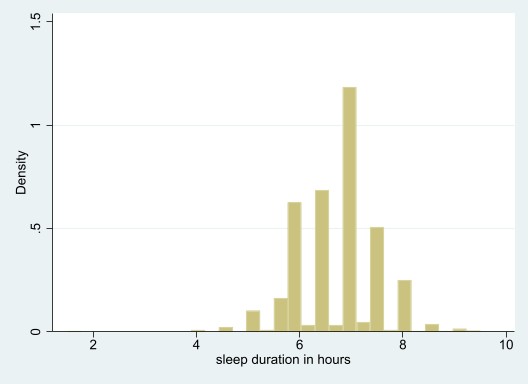


## histogram hours_worked average hours per week


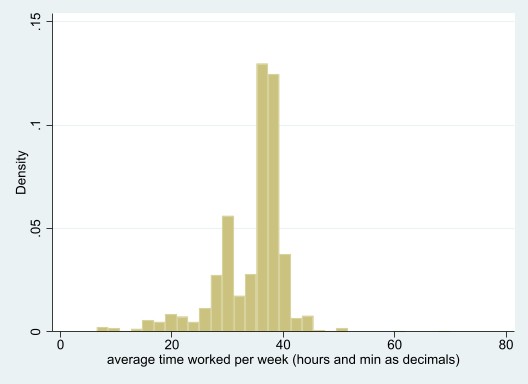


## histogram R10_NetterSisteÅr


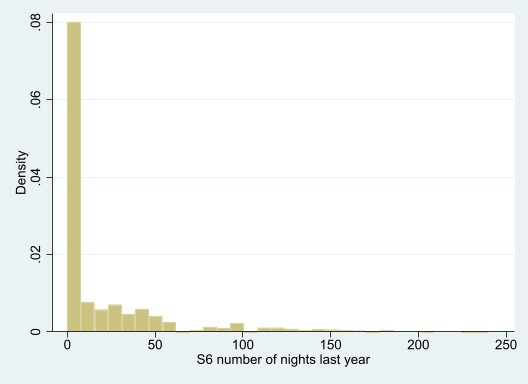


## histogram R10_Mindre11timerFri


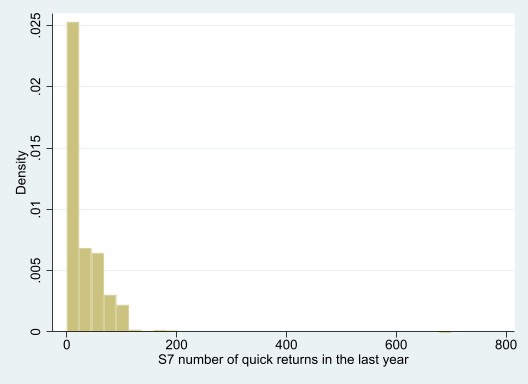


## histogram neck_severity


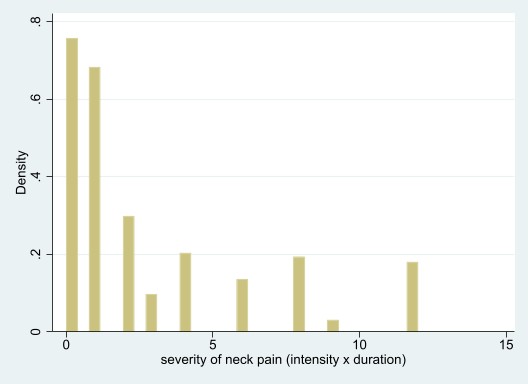


## histogram back_severity


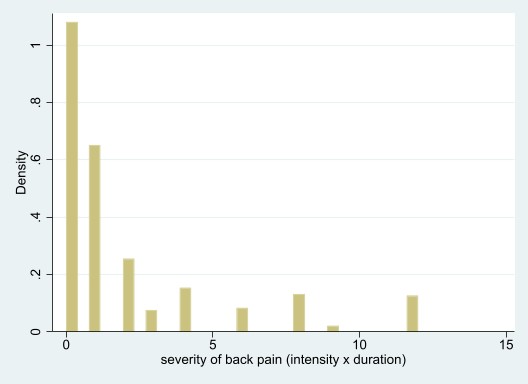


## histogram arm_severity


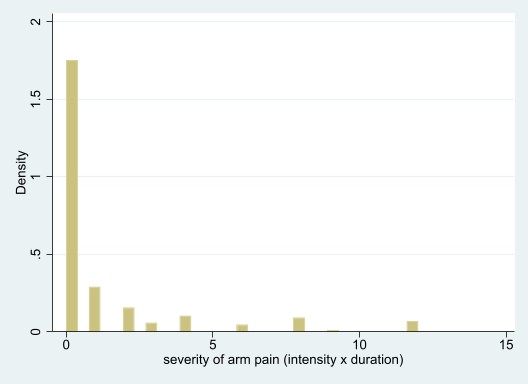


## histogram leg_severity


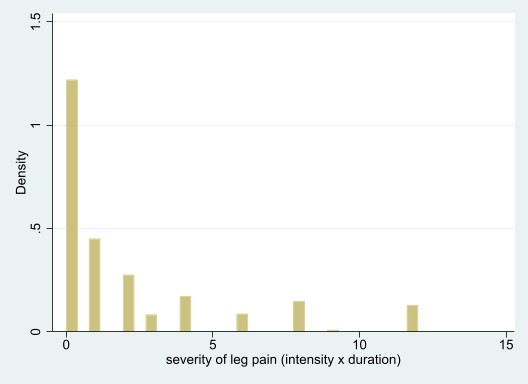


## histogram head_severity


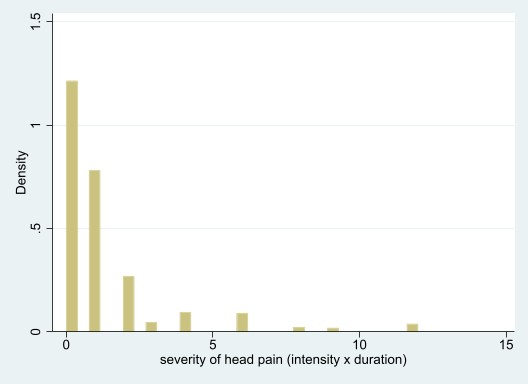


## histogram stomach_severity


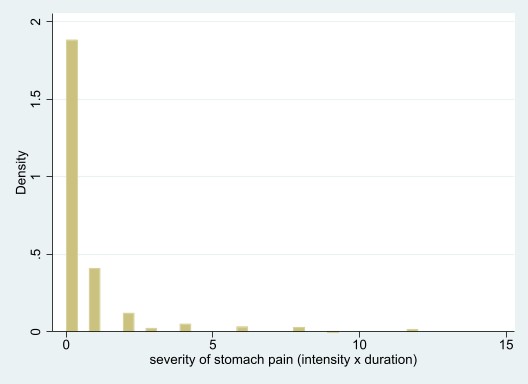


## histogram MSI


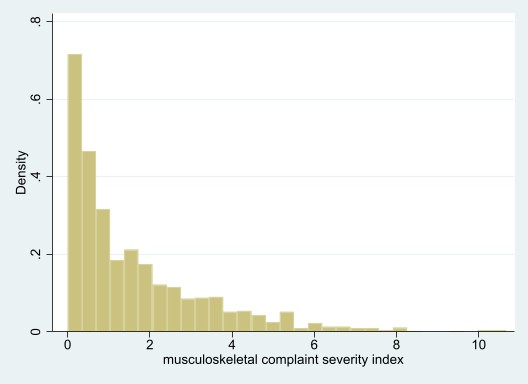


## histogram sum_bis Insomnia Scale


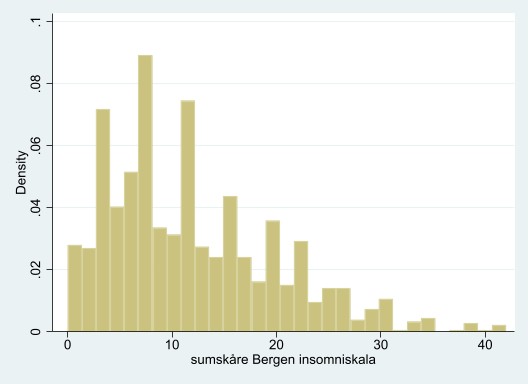


## histogram SUMESS Sleepiness


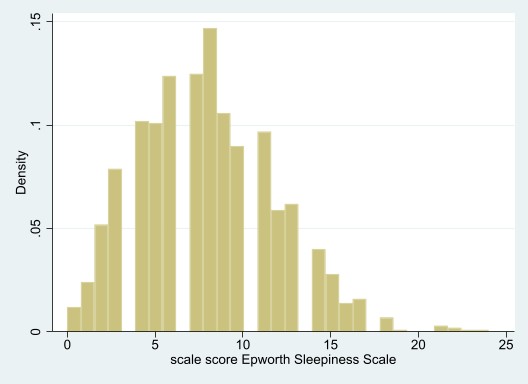


## histogram sumangst Anxiety


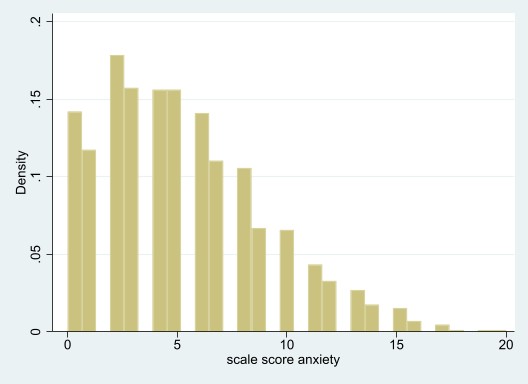


## histogram sumdepr Depression


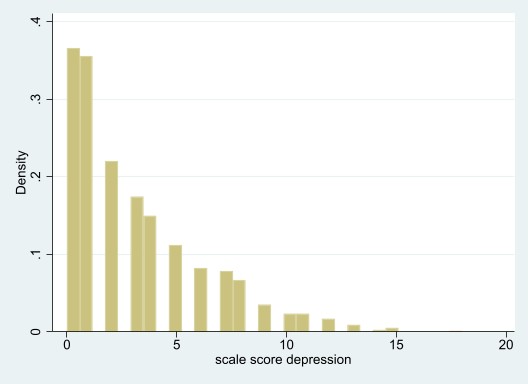


# Linearity Checks ‘crude’

(sex, kids and average hours per week are categorical)

## Age


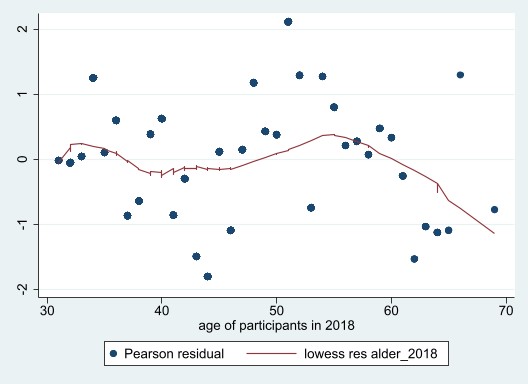


## Sleep duration


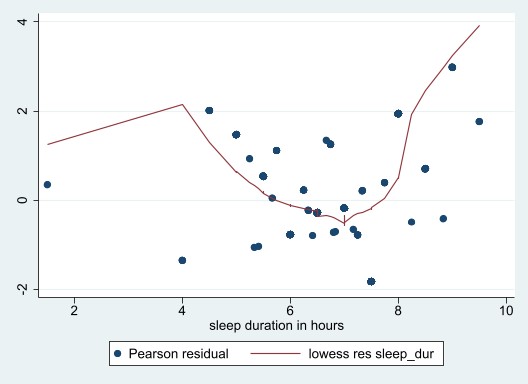


*must use sleep duration as a categorical variable

## hours_worked average hours per week

# MODEL 1 Linearity

## neck_severity


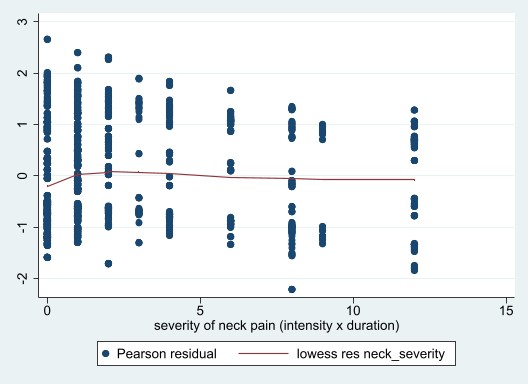


## back_severity


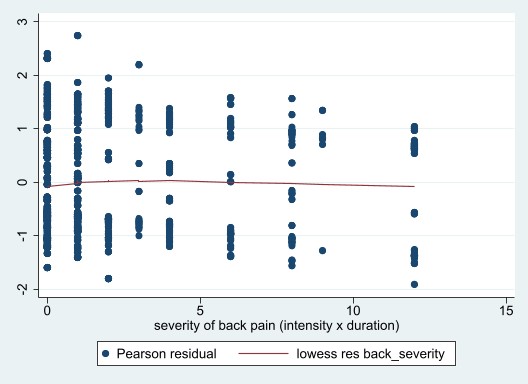


## arm_severity


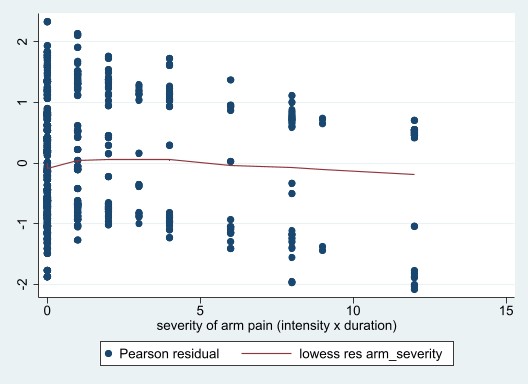


## leg_severity


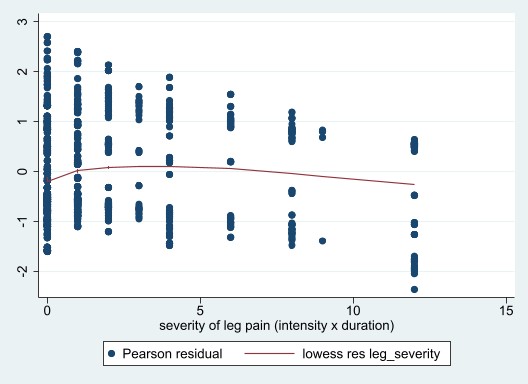


## head_severity


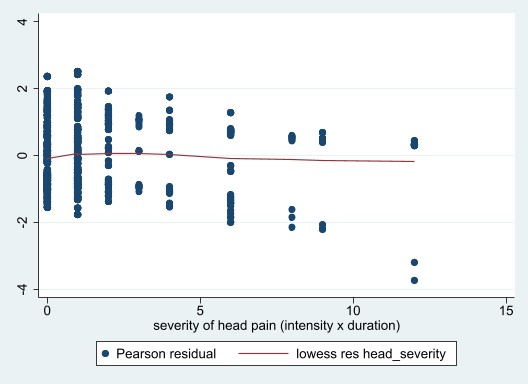


## stomach_severity


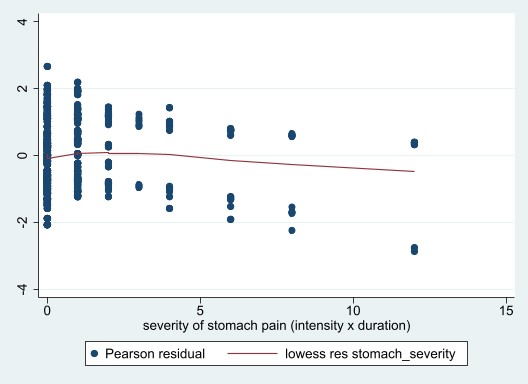


## MSI


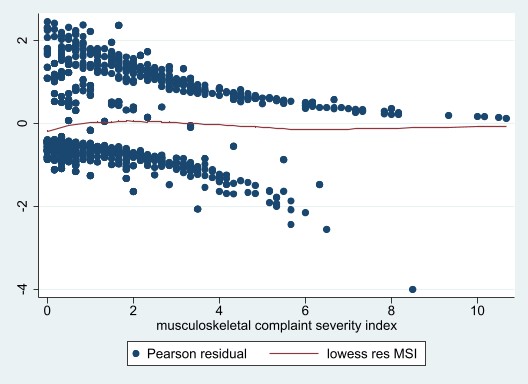


## sum_bis Insomnia Scale


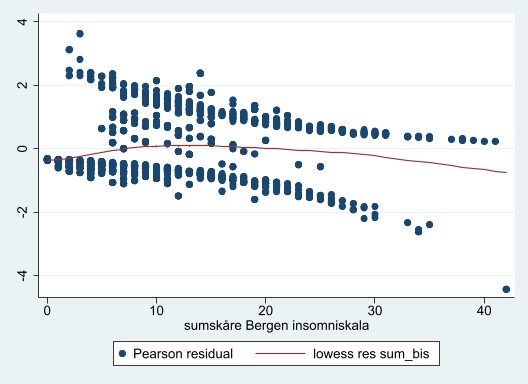


## SUMESS Sleepiness


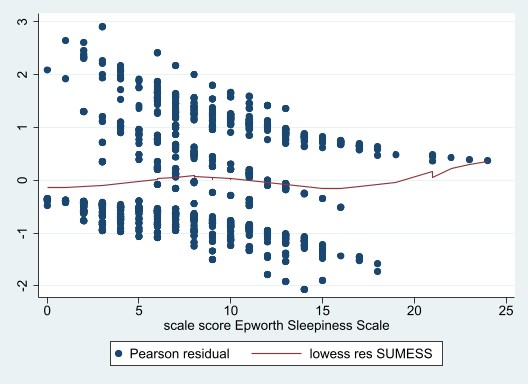


## Sleep duration

#### Continuous


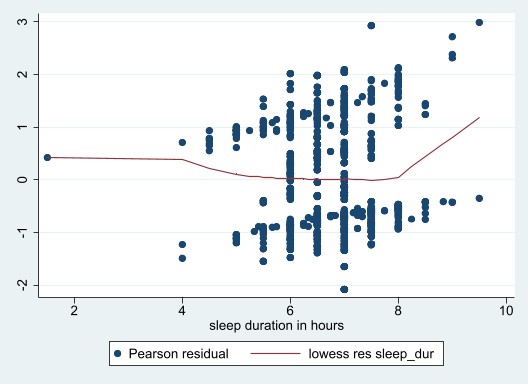


#### Quadratic


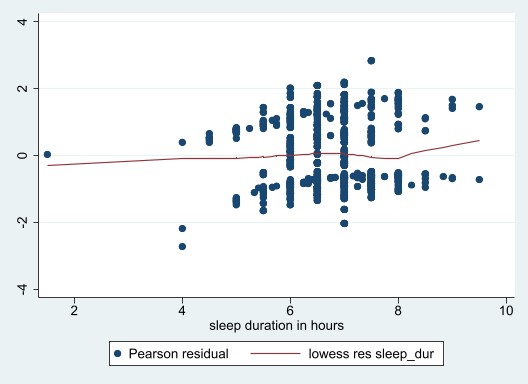


#### Categorical


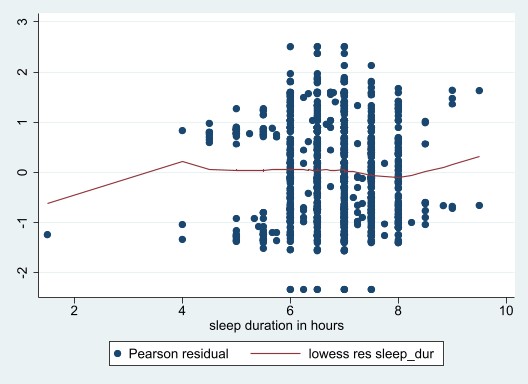


#### Akaike's information criterion and Bayesian information criterion

-----------------------------------------------------------------------------

Model | N ll(null) ll(model) df AIC BIC

-------------+---------------------------------------------------------------

m1(continous) | 1,100 -711.5703 -689.0013 9 1396.003 1441.03

m2 (quadratic) | 1,100 -711.5703 -685.4488 10 1390.898 1440.928

m3 (categorical) | 1,100 -711.5703 -693.6064 10 1407.213 1457.243

Note: BIC uses N = number of observations. See [R] BIC note.

## sumangst Anxiety


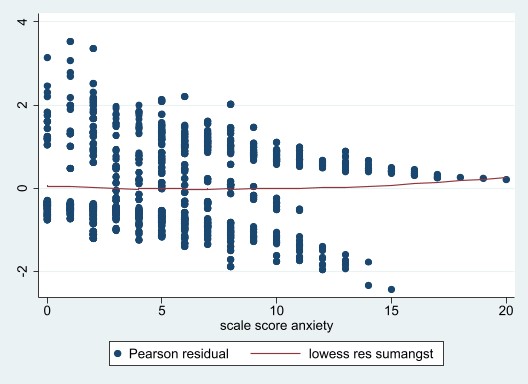


## sumdepr Depression


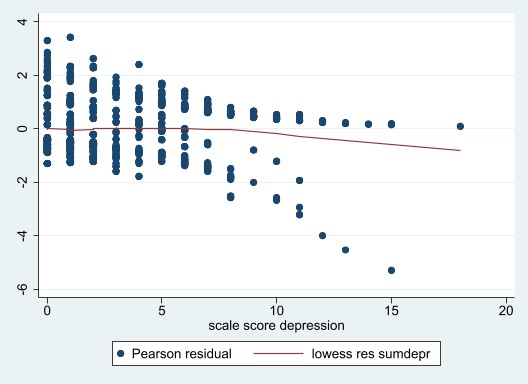


## R10_NetterSisteÅr


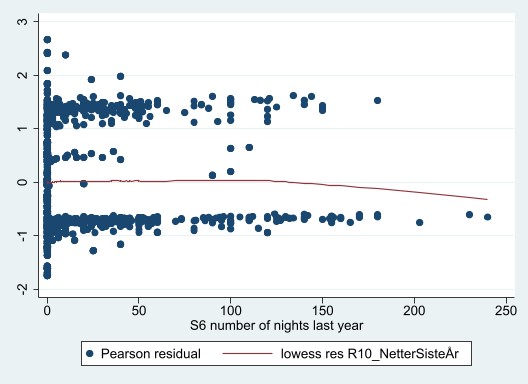


## R10_Mindre11timerFri vs QR_trun

#### Continuous R10_Mindre11timerFri


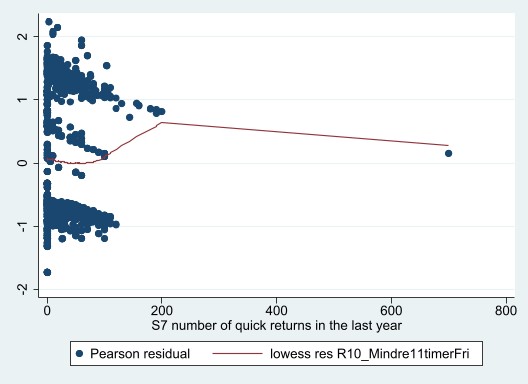


#### Continous QR_trun


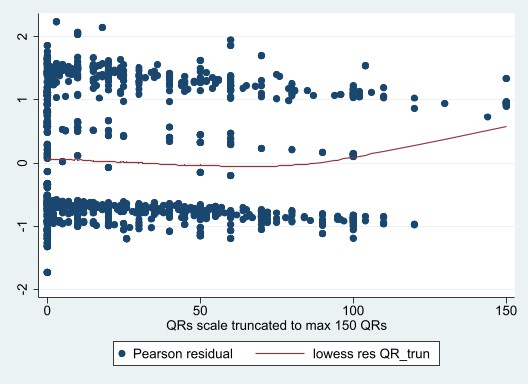


#### Quadratic


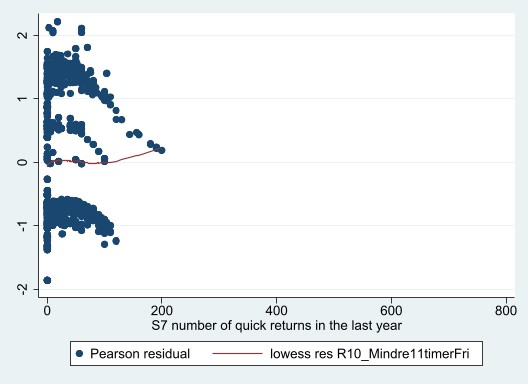


#### Categorical


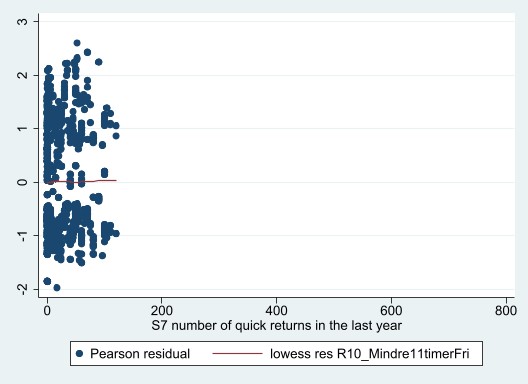


#### Akaike's information criterion and Bayesian information criterion

-----------------------------------------------------------------------------

Model | N ll(null) ll(model) df AIC BIC

-------------+---------------------------------------------------------------

m1 (continuous) | 1,208 -784.6835 -773.5793 9 1565.159 1611.029

m2 (quadratic) | 1,208 -784.6835 -768.4433 10 1556.887 1607.854

m3 (categorical) | 1,124 -734.073 -698.4691 52 1500.938 1762.22

-----------------------------------------------------------------------------

Note: BIC uses N = number of observations. See [R] BIC note.

# MODEL 2 Linearity


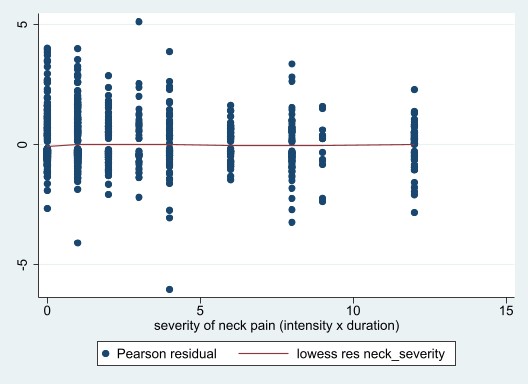


# Model 2 with truncated QR variable


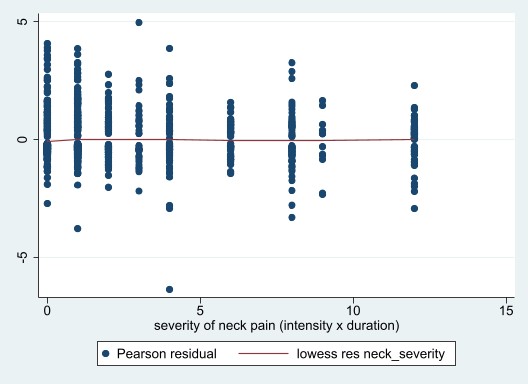

Supplement: S3 File — (DOCX) [file pone.0282734.s003.docx]
